# Formation of Nitric Oxide by Aldehyde Dehydrogenase-2 Is Necessary and Sufficient for Vascular Bioactivation of Nitroglycerin*[Fn FN2]

**DOI:** 10.1074/jbc.M116.752071

**Published:** 2016-09-27

**Authors:** Marissa Opelt, Emrah Eroglu, Markus Waldeck-Weiermair, Michael Russwurm, Doris Koesling, Roland Malli, Wolfgang F. Graier, John T. Fassett, Astrid Schrammel, Bernd Mayer

**Affiliations:** From the ‡ Institute of Pharmaceutical Sciences, Department of Pharmacology and Toxicology, University of Graz, A-8010 Graz, Austria,; the § Institute of Molecular Biology and Biochemistry, Center of Molecular Medicine, Medical University of Graz, 8010 Graz, Austria, and; the ¶ Department of Pharmacology and Toxicology, Ruhr University Bochum, 44780 Bochum, Germany

**Keywords:** cyclic GMP (cGMP), metabolism, mutagenesis in vitro, nitric oxide, vascular smooth muscle cells, aldehyde dehydrogenase-2, nitroglycerin, site-directed mutagenesis

## Abstract

Aldehyde dehydrogenase-2 (ALDH2) catalyzes vascular bioactivation of the antianginal drug nitroglycerin (GTN), resulting in activation of soluble guanylate cyclase (sGC) and cGMP-mediated vasodilation. We have previously shown that a minor reaction of ALDH2-catalyzed GTN bioconversion, accounting for about 5% of the main clearance-based turnover yielding inorganic nitrite, results in direct NO formation and concluded that this minor pathway could provide the link between vascular GTN metabolism and activation of sGC. However, lack of detectable NO at therapeutically relevant GTN concentrations (≤1 μm) in vascular tissue called into question the biological significance of NO formation by purified ALDH2. We addressed this issue and used a novel, highly sensitive genetically encoded fluorescent NO probe (geNOp) to visualize intracellular NO formation at low GTN concentrations (≤1 μm) in cultured vascular smooth muscle cells (VSMC) expressing an ALDH2 mutant that reduces GTN to NO but lacks clearance-based GTN denitration activity. NO formation was compared with GTN-induced activation of sGC. The addition of 1 μm GTN to VSMC expressing either wild-type or C301S/C303S ALDH2 resulted in pronounced intracellular NO elevation, with maximal concentrations of 7 and 17 nm, respectively. Formation of GTN-derived NO correlated well with activation of purified sGC in VSMC lysates and cGMP accumulation in intact porcine aortic endothelial cells infected with wild-type or mutant ALDH2. Formation of NO and cGMP accumulation were inhibited by ALDH inhibitors chloral hydrate and daidzin. The present study demonstrates that ALDH2-catalyzed NO formation is necessary and sufficient for GTN bioactivation in VSMC.

## Introduction

The antianginal drug nitroglycerin (GTN)^2^ causes vasodilation through activation of soluble guanylate cyclase (sGC), resulting in accumulation of cGMP in vascular smooth muscle. The enzymatic pathway underlying GTN bioactivation has remained elusive for several decades. In 2002, Stamler and co-workers (1) proposed aldehyde dehydrogenase-2 (ALDH2) as the key enzyme in GTN bioactivation, and there is general agreement that ALDH2 catalyzes the high affinity pathway of GTN-induced relaxation in rodent and human blood vessels (2, 3). In their initial study, Stamler and colleagues (1) showed that ALDH2 reduces GTN to 1,2-glycerol dinitrate (1,2-GDN) and inorganic nitrite and proposed that nitrite is reduced to NO by components of the mitochondrial respiratory chain. However, we found that GTN activates sGC in the presence of purified ALDH2 (4) and later demonstrated that a minor reaction of ALDH2-catalyzed GTN bioconversion, accounting for about 5% of total turnover, results in direct formation of NO (5–7).

Based on these results, we concluded that ALDH2-catalyzed NO formation explains GTN-induced relaxation of vascular smooth muscle. Although seemingly convincing, this conclusion is called into question by the persistent failure of several independent laboratories to detect the release of NO in vascular tissue and cells exposed to therapeutically relevant low concentrations of GTN (≤1 μm). Measuring oxy- to methemoglobin conversion, Feelisch *et al.* (8) demonstrated for the first time that vascular smooth muscle cells (VSMC) are able to convert GTN into NO, although detection of NO required fairly high concentrations of the nitrate (≥10 μm). Similar results were obtained by Marks *et al.* (9) with bovine pulmonary arteries. Using electron spin resonance (10), NO chemiluminescence (11), and an electrochemical sensor applied intraluminally into rat blood vessels (12), it was later demonstrated that acetylcholine and NO donors give rise to detectable NO signals at concentrations producing vascular relaxation, whereas GTN does not. These data were taken as evidence that vascular relaxation to submicromolar GTN is mediated by an activator of sGC with NO-like bioactivity but not by the free NO radical.

In view of the essential role of ALDH2 in the high affinity pathway of GTN bioactivation, these earlier results might suggest that the minor NO pathway that we discovered is a biologically irrelevant peculiarity of purified ALDH2. To address this issue, we took advantage of a recently developed fluorescent protein-based NO probe referred to as a cyan fluorescent genetically encoded fluorescent NO probe (C-geNOp) that enables real-time monitoring with high spatial and temporal resolution of NO fluctuations on the level of individual cells (13). Generally, geNOps are genetically encoded chimera consisting of a NO-sensitive domain (GAF) that contains a non-heme iron(II) binding domain, which is conjugated to a fluorescent protein. The NO-sensing mechanism is based on a fluorescence quenching phenomenon, which occurs upon NO binding to the probe (13). To specifically investigate the ALDH2-dependent GTN biotransformation in VSMC, the expression of either wild-type ALDH2 or the C301S/C303S mutant was accomplished by adenoviral transfection. We have previously shown that mutation of these cysteine residues results in an almost complete loss of clearance-based GTN denitration, whereas direct reduction of the nitrate to NO is preserved (7). Our results unveiled that cells expressing wild-type ALDH2 or the C301S/C303S mutant rapidly increase cellular NO levels in response to therapeutically relevant concentrations of GTN. The GTN-dependent NO elevation was significantly higher in cells expressing the mutated ALDH2. In addition, the essential role of direct NO formation in GTN bioactivation was demonstrated by enhanced activation of purified sGC in VSMC lysates and cGMP accumulation in intact porcine aortic endothelial cells, which retain sGC expression in culture (14, 15). Taken together, the present study demonstrates that GTN-derived NO signals in single formation fully account for vascular sGC activation by GTN.

## Results

### 

#### 

##### ALDH2 Expression in Non-infected and Infected Cells

**FIGURE 1.**
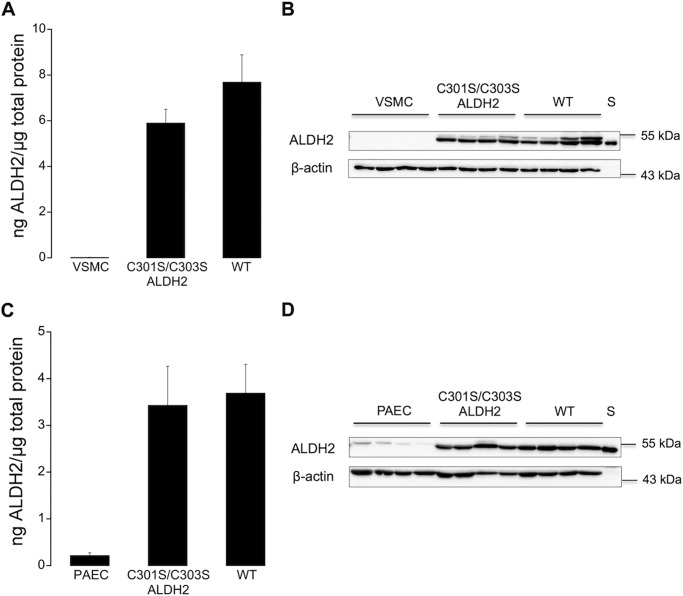
**ALDH2 expression in non-infected and infected vascular smooth muscle and porcine aortic endothelial cells.** *A*, expression of ALDH2 in non-infected and infected (WT or C301S/C303S ALDH2) VSMC (*n* = 4). *B*, representative Western blot of total homogenates of vascular smooth muscle cells (10 μg of protein), showing ALDH2 (54 kDa) and β-actin (43 kDa). Purified human ALDH2 (25 ng) was used as standard (*S*) for protein quantification. *C*, expression of ALDH2 in non-infected and infected (WT or C301S/C303S ALDH2) porcine aortic endothelial cells (*PAEC*) (*n* = 5). *D*, representative Western blot of total homogenates of porcine endothelial cells (10 μg of protein), showing ALDH2 (54 kDa) and β-actin (43 kDa). Purified human ALDH2 (25 ng) was used as standard (*S*) for protein quantification. Summary data are expressed as ng of ALDH2/μg of total protein and represent mean values ± S.E. of the number of experiments indicated in the panel description above.

Protein expression of WT and C301S/C303S ALDH2 after adenoviral overexpression was quantified by immunoblotting using human ALDH2 as a standard. As shown in Fig. 1*A*, infection of ALDH2-deficient VSMC with WT or C301S/C303S ALDH2 resulted in similar protein expression levels of 7.7 ± 1.2 and 5.9 ± 0.6 ng of ALDH2/μg of total protein for wild-type and mutated ALDH2, respectively. ALDH2 was weakly detectable in non-infected VSMC. A representative Western blot is shown in Fig. 1*B*. As shown in Fig. 1*C*, virtually identical results were obtained with porcine aortic endothelial cells (3.7 ± 0.6 and 3.4 ± 0.8 ng of ALDH2/μg of total protein for WT and C301S/C303S ALDH2, respectively). Unlike VSMC, non-infected porcine aortic endothelial cells expressed small amounts of endogenous ALDH2 (0.22 ± 0.06 ng of ALDH2/μg of total protein). A representative Western blot is shown in Fig. 1*D*.

##### Imaging of Cellular GTN-derived NO Signals in VSMC

**FIGURE 2.**
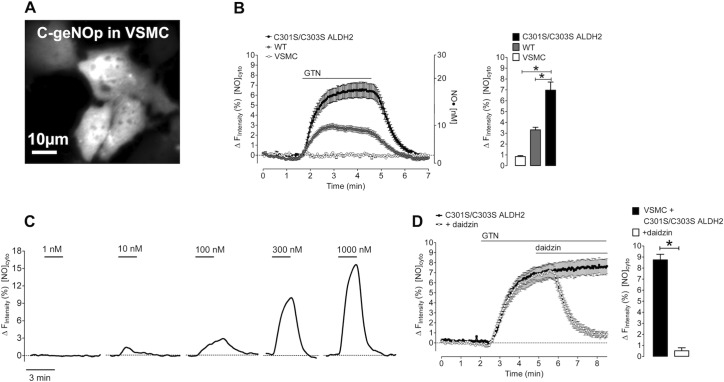
**Live-cell imaging of GTN-derived NO formation in vascular smooth muscle cells.** *A*, representative image of VSMC expressing C-geNOp. *B*, NO release over time (*left panel*) and maximal NO increase (*right panel*) were measured in VSMC infected with C-geNOp alone or in combination with either WT or C301S/C303S ALDH2 in response to 1 μm GTN in the presence of 1 mm DTT by live-cell imaging as described under “Experimental Procedures.” Points represent average values ± S.E. (*n* = 27 for VSMC; *n* = 26 for VSMC+WT; *n* = 20 for VSMC+C301S/C303S ALDH2). Individual traces are shown in supplemental Fig. 1*A*. *[NO]^cyto^*, free NO concentration. *C*, representative curves showing NO release in response to cumulative increasing GTN concentrations (10–1000 nm) in VSMC expressing C301S/C303S ALDH2. Each concentration was administered for 3 min, followed by a washout step. *D*, average curve showing the effect of 0.2 mm daidzin on NO release over time (*left panel*) and maximal NO increase (*right panel*) in VSMC expressing C301S/C303S ALDH2 in response to 1 μm GTN in the presence of 1 mm DTT (*n* = 16 for control, *n* = 19 with daidzin). Data are expressed as inverted curves (1 − *F*/*F*_0_ in %) of the number of experiments indicated in the panel description above (*, *p* < 0.05).

**FIGURE 3.**
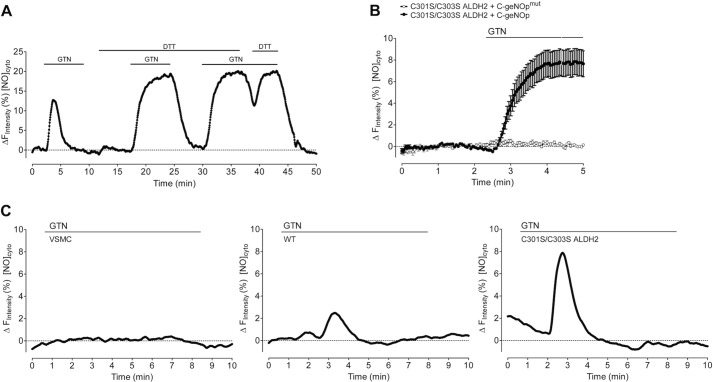
**Live-cell imaging of GTN-derived NO formation in the absence of DTT and determination of the specificity of the NO sensor using C-geNOp^mut^.***A*, representative curve showing the effect of 1 mm DTT on intracellular NO dynamics in VSMC expressing C301S/C303S ALDH2 in response to 1 μm GTN. *[NO]^cyto^*, free NO concentration. *B*, average curves showing fluorescence over time of C-geNOp (*n* = 12) and C-geNOp^mut^ (*n* = 10) upon the addition of 1 μm GTN to vascular smooth muscle cells expressing C301S/C303S ALDH2. Data represent average values ± S.E. *C*, representative curves showing NO release over time in VSMC infected with C-geNOp alone (*left panel*) or in combination with either WT (*middle panel*) or C301S/C303S ALDH2 (*right panel*) in response to 1 μm GTN in the absence of DTT. Data are expressed as inverted curves (1 − *F*/*F*_0_ in %) of the number of experiments indicated in the panel description above.

Formation of GTN-derived NO was measured in single VSMC expressing C-geNOp, the cyan fluorescent genetically encoded NO probe (Fig. 2*A*). As shown in Fig. 2*B*, the addition of 1 μm GTN to cells expressing either wild-type or mutated ALDH2 rapidly increased intracellular NO levels (individual traces are shown in supplemental Fig. 1*A*), whereas VSMC expressing C-geNOp only did not respond to GTN addition (baseline in Fig. 2*B*). ALDH2-catalyzed formation of GTN-derived NO was biphasic with a rapid initial phase within the first minute of GTN administration, followed by a stable plateau phase until GTN washout (Fig. 2*B*). The initial rate of NO formation was 2-fold higher in the presence of C301S/C303S ALDH2 when compared with WT-infected cells (0.80 ± 0.09 and 0.41 ± 0.02 Δ*F* min^−1^, respectively). The estimated maximal concentrations of NO generated from 1 μm GTN were ∼7 and ∼17 nm for wild-type and mutated ALDH2, respectively. ALDH2-catalyzed formation of GTN-derived NO is about 25% (WT) and 55% (C301S/C303S ALDH2) of maximal NO release from 3-(2-hydroxy-1-methyl-2-nitrosohydrazino)-*N*-methyl-1-propanamine (NOC-7) (see supplemental Fig. 1*B*). In VSMC expressing C301S/C303S ALDH2, NO formation was detectable at submicromolar concentrations (10 nm to 1 μm) of the nitrate (Fig. 2*C*). As shown in Fig. 2*D*, the ALDH2 inhibitor daidzin (0.2 mm) completely inhibited GTN-derived NO formation in cells expressing C301S/C303S ALDH2 in a rapid and reversible manner (supplemental Fig. 2). All experiments described above, performed in the presence of 1 mm DTT, showed a rapid initial phase of NO formation, followed by a stable plateau upon GTN application. As shown in Fig. 3*A*, in the absence of DTT, the addition of 1 μm GTN to cells expressing C301S/C303S ALDH2 resulted in a transient NO signal. Washout of DTT during the stable plateau phase led to an immediate decrease of the NO signal that was restored by the re-addition of DTT. Transient NO production in the absence of DTT was also observed in cells expressing WT ALDH2 (Fig. 3*C*). Specificity of the sensor was confirmed using the NO-insensitive C-geNOp^mut^that is not able to bind NO. In contrast to functional C-geNOp, the fluorescence signal of the NO-insensitive probe remained unaffected by the addition of NOC-7 (13) or 1 μm GTN in VSMC expressing C301S/C303S ALDH2 (Fig. 3*B*).

##### GTN-induced Activation of Purified sGC by Infected VSMC Lysates

**FIGURE 4.**
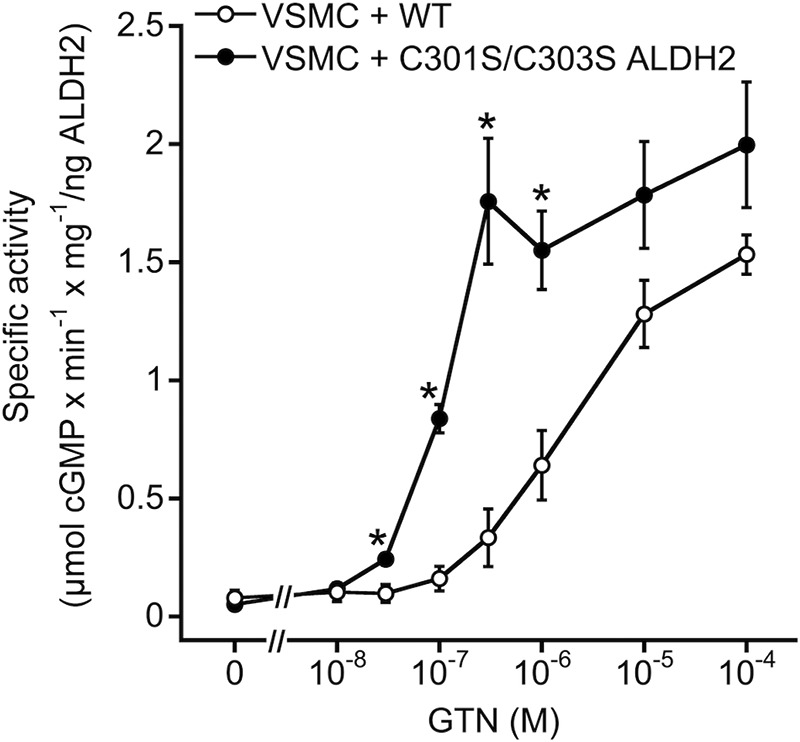
**GTN-triggered activation of purified sGC in the presence of vascular smooth muscle cell lysates.** 150 μg of cell lysates from VSMC expressing either WT or C301S/C303S ALDH2 were incubated at 37 °C for 10 min in the presence of 50 ng of sGC, [α-^32^P]GTP (∼200,000 cpm), 1,000 units/ml SOD, 1 mm NAD^+^, 2 mm DTT, and the indicated concentrations of GTN (10 nm to 100 μm), followed by isolation and quantification of cGMP as described under “Experimental Procedures.” Data were normalized to ALDH2 content to account for different expression levels and represent mean values ± S.E. of 3–4 independent experiments (*, *p* < 0.05).

Activation of purified sGC by increasing GTN concentrations (10 nm to 100 μm) was determined with lysates prepared from VSMC expressing either wild-type or mutated ALDH2. As shown in Fig. 4, GTN caused concentration-dependent sGC activation with EC_50_ values of 2.87 ± 0.68 and 0.11 ± 0.01 μm in the presence of lysates from WT and C301S/C303S ALDH2-infected cells, respectively, indicating more than 10-fold higher apparent GTN affinity of the mutant. Activation of sGC was not observed at <10 μm GTN in lysates prepared from non-infected cells (data not shown). The effect of higher GTN concentrations was not sensitive to ALDH2 inhibitors and apparently reflects an ALDH2-independent pathway of GTN bioactivation (data not shown).

##### GTN-triggered cGMP Accumulation in Intact Porcine Aortic Endothelial Cells

**FIGURE 5.**
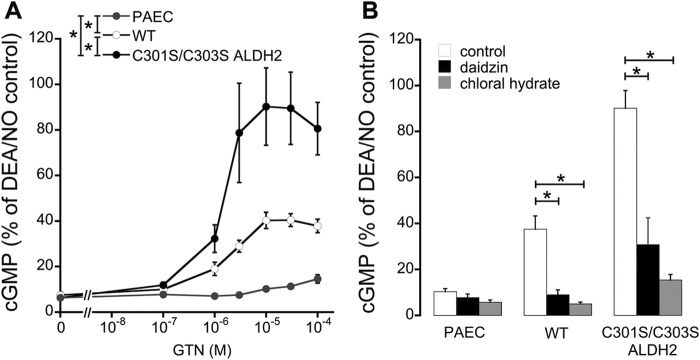
**GTN-induced cGMP accumulation in cultured porcine aortic endothelial cells.** *A*, non-infected and infected (WT or C301S/C303S ALDH2) porcine aortic endothelial cells (*PAEC*) were incubated with the indicated concentrations of GTN (100 nm to 100 μm), and cGMP formation was determined as described under “Experimental Procedures.” *B*, effects of 0.4 mm daidzin and 5 mm chloral hydrate on cellular cGMP accumulation was determined in the presence of 10 μm GTN. Data were normalized to maximal cGMP formation induced by DEA/NO (1 μm) under identical conditions and represent mean values ± S.E. of 3–5 independent experiments (*, *p* < 0.05).

To measure cGMP accumulation in response to different GTN concentrations (100 nm to 100 μm) in intact cells, we overexpressed wild-type and mutated ALDH2 in porcine aortic endothelial cells, which retain sGC expression during culture (15). As shown in Fig. 5*A*, GTN caused concentration-dependent cGMP accumulation in cells expressing either wild-type or mutated ALDH2 with maximal cGMP levels of 40 ± 3% and 93 ± 15% of 2,2-diethyl-1-nitroso-oxyhydrazine (DEA/NO) (1 μm) for WT and C301S/C303S ALDH2, respectively. As illustrated in Fig. 5*B*, preincubation with established ALDH inhibitors daidzin (0.4 mm) and chloral hydrate (5 mm) caused pronounced inhibition of GTN-induced cGMP formation. DEA/NO-induced cGMP accumulation was not significantly affected by ALDH2 overexpression (supplemental Fig. 3).

##### GTN Denitration

**FIGURE 6.**
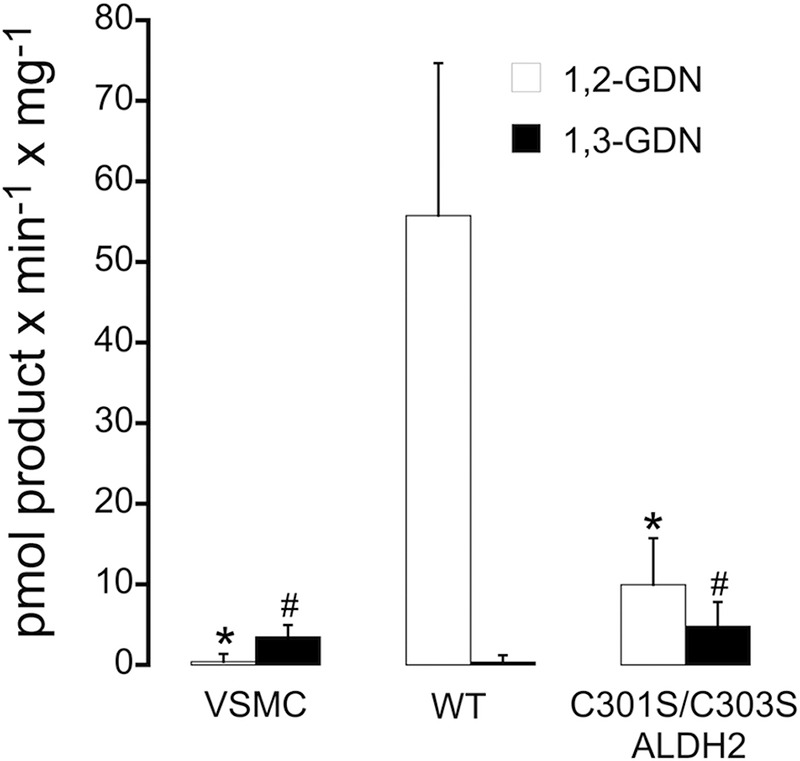
**GTN denitration activity of vascular smooth muscle cell lysates.** 300 μg of cell lysates from non-infected or infected VSMC (WT or C301S/C303S ALDH2) were incubated with 2 μm ^14^C-labeled GTN at 37 °C for 10 min in a final volume of 0.2 ml of 50 mm potassium phosphate buffer, pH 7.4, containing 3 mm MgCl_2_, 2 mm EDTA, 1 mm NAD^+^, 0.1 mm DTPA, and 2 mm DTT. Reaction products were extracted and quantified by radio thin-layer chromatography. Data represent mean values ± S.E. of 4 independent experiments (*, *p* < 0.05 *versus* 1,2-GDN formation catalyzed by WT ALDH2-infected cell lysates; #, *p* < 0.05 *versus* 1,3-GDN formation catalyzed by WT ALDH2-infected cell lysates).

As shown in Fig. 6, the rates of 1,2- and 1,3-GDN formation were 0.40 ± 0.10 and 3.5 ± 1.5 pmol min^−1^ mg^−1^, respectively, in lysates prepared from non-infected VSMC, demonstrating the virtual lack of specific GTN denitration in the absence of ALDH2 expression. In VSMC lysates expressing WT ALDH2, 1,2-GDN formation was increased to 55.8 ± 18.9 pmol min^−1^ mg^−1^, whereas the 1,3-isomer was weakly detectable (0.35 ± 0.1 pmol min^−1^ mg^−1^). Expression of the C301S/C303S mutant led to markedly reduced rates of 1,2-GDN formation (9.9 ± 5.8 pmol min^−1^ mg^−1^) that were accompanied by significantly increased formation of the 1,3-isomer (4.8 ± 3.0 pmol min^−1^ mg^−1^). Similar results were obtained with lysates prepared from porcine aortic endothelial cells expressing either WT or C301S/C303S ALDH2 (data not shown).

## Discussion

It is generally accepted that ALDH2 is essential for GTN bioactivation in rodent and human blood vessels, but the underlying mechanism is still unclear. In particular, the link between ALDH2-catalyzed GTN metabolism, yielding mainly inorganic nitrite, and activation of sGC has remained elusive. Because inorganic nitrite does not activate sGC at biologically relevant concentrations, it has been suggested that NO is formed by the nitrite reductase activity of the mitochondrial respiratory chain and possibly converted to a more stable *S*-nitrosothiol intermediate (1). However, respiratory rate does not affect GTN bioactivation by isolated mitochondria, calling into question an involvement of the respiratory chain (16), and subsequent studies revealed a minor pathway of ALDH2-catalyzed GTN bioconversion that yields free NO radical (4–7). The NO pathway accounts only for about 5% of total turnover catalyzed by purified ALDH2, but NO formation could explain GTN bioactivation without invoking a mysterious link to sGC activation. This concept of vascular GTN bioactivation appears to be conclusive but suffers from lack of evidence in vascular tissue. In view of the striking failure to correlate NO formation with GTN bioactivity in blood vessels (10–12), testing the NO hypothesis of ALDH2-catalyzed GTN bioactivation in vascular tissue appears to be crucial.

We have previously shown that mutation of the two cysteine residues adjacent to the reactive Cys-302 residue (Cys-301 and Cys-303) results in an ALDH2 variant that demonstrated markedly reduced GTN denitration activity but retained NO formation (7). In fact, the purified mutant turned out to exhibit significantly higher GTN affinity and initial rates of NO formation, and generated about 2-fold higher maximal concentrations of NO than the WT enzyme (7). Thus, overexpression of this mutant in cells should conclusively clarify whether or not the NO pathway of the ALDH2 reaction is sufficient for vascular GTN bioactivation.

In the present study, we expressed the C301S/C303S double mutant in endothelial cells and VSMC and correlated GTN-triggered activation of sGC with intracellular NO formation. Intracellular NO measurements were made possible with a novel, highly sensitive C-geNOp that responds dynamically and specifically in a concentration-dependent manner to NO in single cells (13). Specificity of the signal was confirmed with a mutated sensor that does not bind NO and does not respond to GTN. GTN was reported to cause formation of superoxide in vascular tissue, raising the concern that H_2_O_2_ could lead to artifacts during NO imaging. However, H_2_O_2_ at physiological concentrations does not affect the responsiveness of geNOps because they are highly selective to NO (13). This enables a clear discrimination of real NO signals from other reactive oxygen species including H_2_O_2_, O_2_^−^, and ONOO^−^ (13). Thus, we observed no effects of SOD and catalase on the GTN response of isolated rodent blood vessels or cultured cells (data not shown), indicating that GTN does not cause significant oxidative stress in our experimental models.

The strong signal observed upon expression of the C301S/C303S mutant shows that ALDH2-catalyzed bioconversion of GTN in VSMC is associated with formation of NO without significant contribution of the major clearance-based reaction pathway. It should be emphasized that nanomolar concentrations of GTN gave rise to significant formation of NO. This is an essential point because relaxation of rodent blood vessels to GTN is biphasic (17, 18), indicating the contribution of high and low affinity pathways. Although the high affinity pathway, caused by up to 1 μm GTN, is sensitive to ALDH2 inhibitors and absent in ALDH2 knock-out mice, the low affinity pathway observed at higher GTN concentrations is ALDH2-independent (19, 20), possibly due to formation of *S*-nitrosated intermediates (21) that activate sGC through the release of NO (22). Response of the sensor to nanomolar GTN shows that NO mediates vascular bioactivation of therapeutically relevant low concentrations of GTN.

GTN-induced increase in cellular NO correlated well with activation of purified sGC in the presence of VSMC lysates. Significant increases in sGC activity were observed with 30 and 300 nm GTN in cells that had been infected with C301S/C303S and WT ALDH2, respectively, confirming the ∼10-fold higher affinity of the mutant we reported previously (7). We observed that VSMC isolated from rodent blood vessels rapidly lost sGC expression in culture as described previously (data not shown) (14). Therefore, we could not test for accumulation of cGMP in cultured VSMC and instead used porcine aortic endothelial cells, which retain sGC expression in culture. Overexpression of WT and C301S/C303S ALDH2 caused pronounced accumulation of cGMP in response to GTN. The maximal cGMP levels measured in C301S/C303S ALDH2-infected cells were similar to the effect of the established NO donor DEA/NO. In endothelial cells, the GTN concentration-response curve was rightward-shifted when compared with activation of sGC in the presence of VSMC lysates. However, the experimental setups (cell-free *versus* intact cells) are hardly comparable, and competing reactions of GTN clearance (23) and/or NO scavenging (24) may be limiting. As GTN-induced relaxation of isolated blood vessels is enhanced by the removal of the endothelium (25, 26), inhibition of endothelium-dependent relaxation (27, 28), or knock-out of endothelial NO synthase (29), it is also conceivable that cGMP formation was impaired by basal NO formation.

There are numerous studies on an apparent discrepancy between the GTN concentration response in terms of blood vessel relaxation and accumulation of tissue cGMP. We also found that the EC_50_ of GTN necessary to cause formation of NO in cultured cells or cell-free preparations is about 10-fold higher than its vasodilatory potency (6). However, this comparison is misleading because an increase in cGMP levels to less than 5% of the maximal effect caused by NO donors is sufficient for 100% vasorelaxation, indicating that blood vessels contain a large fraction of spare sGC NO receptor (4, 30). Thus, the GTN affinity of NO formation measured with the NO sensor in ALDH2-transfected cells appears to sufficiently explain GTN-induced vasodilation.

The observation that expression of C301S/C303S ALDH2 led to cGMP accumulation in response to low GTN is a crucial finding of the present study. Enhanced formation of NO and markedly decreased rates of 1,2-GDN formation confirm that mutation of the two cysteine residues causes a pronounced shift from clearance-based GTN metabolism, yielding inorganic nitrite, to the minor NO pathway that accounts for about 5% of total turnover. Thus, our data conclusively show that the NO pathway of ALDH2 catalysis explains bioactivation of clinically relevant low concentrations of GTN.

Prolonged administration of GTN results in the development of nitrate tolerance, presumably due to mechanism-based oxidative inactivation of ALDH2 in the course of GTN turnover (31). *In vitro*, ALDH2 inactivation is partially overcome by high concentrations of reductants such as DTT (32) or dihydrolipoic acid (33), but the mechanism of ALDH2 regeneration in tissues is still unknown. Our observations of transient NO production with both WT and C301S/C303S ALDH2 in the absence of DTT and immediate decrease of the NO signal upon washout of the reductant support the view that ALDH2 inactivation occurs through oxidation of the catalytically active Cys-302 residue. The essential requirement of a reductant for sustained formation of GTN-derived NO in VSMC contrasts with the comparably long-lasting action of the nitrate *in vivo* (hours) or in isolated blood vessels (minutes), suggesting the loss of a protective agent in the course of VSMC isolation and culture. However, further work is required to clarify the mechanism underlying ALDH2 reactivation in blood vessels.

The present results combined with our previous studies using purified WT and mutated ALDH2 (7) clearly demonstrate that free NO radical mediates the GTN-induced activation of sGC in the vasculature. This raises the question of why several independent laboratories consistently failed to detect GTN-derived NO in blood vessels exposed to biologically relevant concentrations of GTN (10–12). Assuming colocalization of ALDH2 and sGC in vascular smooth muscle, it is conceivable that NO escapes detection by close interaction of ALDH2 with sGC in a spatially confined manner, whereas NO is released globally by agonists such as acetylcholine or NO donor compounds. To address this issue, we are currently studying the subcellular localization of ALDH2 and sGC by immunogold labeling and scanning electron microscopy. In addition, we are trying to modify sensor-based NO detection to enable measurements of cellular NO dynamics in intact blood vessels using the geNOps technology.

## Experimental Procedures

### 

#### 

##### Materials

Bovine lung sGC was purified as described previously (34). Human ALDH2 was expressed in *Escherichia coli* BL21 (DE3) and purified as described (5, 35). Protein concentrations are expressed per monomer, assuming a molecular mass of 54 kDa. EDTA-free Complete^TM^ Protease Inhibitor Cocktail Tablets were from Roche Diagnostics GmbH (Vienna, Austria). Pierce^TM^ BCA Protein Assay Kit was obtained from Fisher Scientific Austria GmbH (Vienna, Austria). [α-^32^P]GTP (800 Ci/mmol) was obtained from PerkinElmer Life Sciences (Vienna, Austria). [2-^14^C]GTN (50 mCi/mmol) was from American Radiolabeled Compounds, purchased through Hartmann Analytic GmbH (Braunschweig, Germany). Nitro POHL® ampoules (G. Pohl-Boskamp GmbH & Co., Hohenlockstedt, Germany), containing 4.4 mm GTN in 250 mm glucose, were obtained from a local pharmacy and diluted with distilled water. Unlabeled organic nitrates used as standards in radio thin-layer chromatography (GTN, 1,2-GDN, and 1,3-GDN) were obtained from LGC Standards (Wesel, Germany). DEA/NO was from Enzo Life Sciences (Lausen, Switzerland) and purchased through Eubio (Vienna, Austria). DEA/NO was dissolved and diluted in 10 mm NaOH. NOC-7 was obtained from Santa Cruz Biotechnology (Santa Cruz, CA). NOC-7 was dissolved and diluted in distilled water. Antibiotics and fetal calf serum were obtained from PAA Laboratories (Linz, Austria). Adeno-X 293 cells were obtained from Takara Bio Europe (Saint-Germain-en-Laye, France). Adenovirus encoding C-geNOp and iron(II)fumarate solution were obtained from NGFI Next Generation Fluorescence Imaging GmbH (Graz, Austria). Chloral hydrate was from Fluka Chemie (Vienna, Austria). Culture medium and all other chemicals were from Sigma-Aldrich (Vienna, Austria). Daidzin was dissolved and diluted in DMSO.

##### Generation of Recombinant Adenoviral Vectors

Mutation of Cys-301 and Cys-303 to serine in human ALDH2 was performed as described (7). Adenoviral vectors encoding human wild-type ALDH2 (WT) or the C301S/C303S mutant (C301S/C303S ALDH2) under the control of the CMV promotor were generated as described (36). All adenoviral vectors were propagated in Adeno-X 293 cells and purified by CsCl ultracentrifugation, and the titer was determined by using the AdEasy Viral Titer Kit (Agilent Technologies, Vienna, Austria).

##### Cell Culture and Adenoviral Infection

VSMC were isolated from ALDH2 KO mice and immortalized as described (36). Porcine aortic endothelial cells were isolated as described previously (15). Cells were cultured in DMEM, supplemented with 10% (v/v) heat-inactivated fetal calf serum, 100 units/ml penicillin, 0.1 mg/ml streptomycin, and 1.25 μg/ml amphotericin in humidified atmosphere (95% O_2_/5% CO_2_) at 37 °C. For adenoviral infection, subconfluent vascular smooth muscle and porcine endothelial cells were incubated with AdV-WT or AdV-C301S/C303S ALDH2 at a multiplicity of infection (MOI) of 10 in DMEM containing 10% fetal calf serum. After 24 h of incubation, medium was removed and cells were incubated at 37 °C for a further 24 h. Non-infected cells were used as control. For single cell NO measurements, cells were infected with the genetically encoded cyan fluorescent NO probe (AdV-CgeNOp) alone at MOI 3 or in combination with either AdV-WT or AdV-C301S/C303S ALDH2, both at MOI 7 in serum-free DMEM. After 1 h of infection, DMEM containing 20% fetal calf serum was added and cells were incubated at 37 °C for 30–48 h. Cellular protein expression was determined by quantitative immunoblotting (see below).

##### Immunoblotting

Adenovirus-infected (WT or C301S/C303S ALDH2) or non-infected cells were harvested and homogenized by sonication (3 × 5 s) in 10 mm Tris buffer, pH 7.4, containing 125 mm potassium chloride, 5 mm EGTA, 2 mm MgCl_2_, and Complete^TM^ Protease Inhibitor Cocktail. Protein concentration was determined with the Pierce^TM^ BCA Protein Assay Kit using bovine serum albumin as standard. Denatured samples (10 μg) were separated by SDS-PAGE on 10% gels and transferred electrophoretically to nitrocellulose membranes. After blocking with 5% nonfat dry milk in PBS containing 0.1% (v/v) Tween 20 for 1 h, membranes were incubated overnight at 4 °C with a primary polyclonal antibody to human ALDH2 (1:20,000; kindly provided by Dr. Henry Weiner) or to β-actin (1:200,000; Sigma). Thereafter, membranes were washed three times and incubated for 1 h with a horseradish peroxidase-conjugated anti-rabbit (ALDH2) or anti-mouse (β-actin) IgG secondary antibody (1:5,000). Immunoreactive bands were visualized by chemiluminescence using an ECL detection reagent (Biozym, Hessisch Oldendorf, Germany) and quantified densitometrically using the Fusion SL system (PEQLAB, VWR International).

##### Single Cell NO Measurements with C-geNOp

GTN-derived NO formation catalyzed by infected VSMC (C-geNOp alone or in combination with either WT or C301S/C303S ALDH2) was determined by live-cell imaging of cells expressing C-geNOp as described recently (13). Prior to fluorescence microscopy, cells were incubated for 10 min with non-toxic iron(II) booster solution, containing 1 mm iron(II) fumarate and 1 mm vitamin C. During the experiments, cells were perfused in the same physiological buffer without iron(II) fumarate and vitamin C, pH 7.4, containing 140 mm NaCl, 5 mm KCl, 2 mm CaCl_2_, 1 mm MgCl_2_, 10 mm d-glucose, and 10 mm HEPES. Intracellular NO release from 10 μm NOC-7 and 1 μm GTN was measured in the absence and presence of 1 mm DTT and 0.2 mm daidzin as indicated in the text and figure legends. NOC-7, GTN, DTT, and daidzin were transiently applied to the cells by using a gravity-based perfusion system. Measurements were performed using a fully automated inverted fluorescent microscope (Till Photonics, Gräfelfing, Germany). C-geNOp was excited at 430 nm via a polychrome V (Till Photonics). Emitted light was collected at 480 nm (emission filter, 482/18 nm; Till Photonics) and visualized using a 20× objective (Zeiss, Göttingen, Germany) and a charge-coupled device (CCD) camera (AVT Stingray F145B, Allied Vision Technologies, Stadtroda, Germany). The fluorescence microscope was controlled using the Live Acquisition 2.0.0.12 software (Till Photonics). Changes of fluorescence intensity of C-geNOp over time were analyzed and plotted in percentage (Δ*F*_Intensity_ %) using the following equation: (1 − *F*/*F*_0_)× 100%, where *F* is the measured fluorescence intensity over time and *F*_0_ is the fluorescence intensity of C-geNOp over time of untreated cells (prior to and after cell treatment) reflecting the bleaching function of the fluorescent probe. Changes in the free NO concentration (Δ[NO]_cyto_) in nm were calculated from the normalized signals using the following equation

(Eq. 1) $${\left[\text{NO}\cdot \right]}_{\text{cyto}}=\frac{\Delta F\times K}{\left(\Delta F_{\max}-\Delta F\right)}$$

as described with *k* = 4.50 and Δ*F*_max_ = 19.26% for C-geNOp (13).

##### Determination of GTN Bioactivation

Purified bovine lung sGC (50 ng) was incubated at 37 °C for 10 min in a final volume of 0.1 ml with increasing concentrations of GTN (10 nm to 100 μm) in the presence of 150 μg of cell lysates from infected VSMC (WT or C301S/C303S ALDH2). Assay mixtures contained 50 mm triethanolamine (pH 7.4), 0.5 mm [α-^32^P]GTP (∼200,000 cpm), 3 mm MgCl_2_, 1 mm cGMP, 1 mm 3-isobutyl-1-methylxanthine, 1 mm EGTA, 2 mm DTT, 0.1 mm DTPA, 5 mm creatine phosphate, 152 IU/I creatine kinase, 1 mm NAD^+^, and 1,000 units/ml SOD. Reactions were terminated by the addition of 0.45 ml of zinc acetate (120 mm) and 0.45 ml of sodium bicarbonate (120 mm). After centrifugation (20,000 × *g* at 4 °C for 10 min), supernatants were applied to aluminum oxide columns, which had been acidified with 100 mm perchloric acid. Columns were washed with distilled water, and [^32^P] was eluted with sodium acetate (50 mm) and quantified by liquid scintillation counting. Blank values were measured in the absence of sGC and subtracted. Data were normalized to ALDH2 content to account for different expression levels.

##### Determination of Cellular cGMP Accumulation

Non-infected or infected porcine aortic endothelial cells (WT or C301S/C303S ALDH2), grown in 24-well plates, were incubated for 15 min at 37 °C in 50 mm Tris buffer, pH 7.4, containing 100 mm NaCl, 5 mm KCl, 1 mm MgCl_2_, 2.5 mm CaCl_2_, 2 mm DTT, 1,000 units/ml SOD, 1 mm 3-isobutyl-1-methylxanthine, 1 μm indomethacin, and where indicated, 5 mm chloral hydrate or 0.4 mm daidzin. Reactions were started by the addition of GTN (100 nm to 100 μm) or DEA/NO (1 μm) and terminated after 10 min by the removal of the incubation medium and the addition of 0.01 n HCl. Within 1 h, intracellular cGMP was completely released into supernatant and measured by radioimmunoassay (15). Data were normalized to maximal cGMP formation induced by DEA/NO under identical conditions.

##### Determination of GTN Denitration by Radio Thin-layer Chromatography

The rates of GTN denitration catalyzed by non-infected or infected vascular smooth muscle and porcine aortic endothelial cells (WT or C301S/C303S ALDH2) were determined as conversion of GTN into 1,2-GDN and 1,3-GDN as described (4). Cells were washed with phosphate-buffered saline, trypsinized, and sonicated (3 × 5 s), and protein concentration was determined with the Pierce^TM^ BCA Protein Assay Kit. Cell lysates (300 μg) were incubated with 2 μm ^14^C-labeled GTN at 37 °C for 10 min in a final volume of 0.2 ml of 50 mm potassium phosphate buffer, pH 7.4, containing 3 mm MgCl_2_, 2 mm EDTA, 1 mm NAD^+^, 0.1 mm DTPA, and 2 mm DTT. GTN and denitrated metabolites were extracted twice with 1 ml of diethyl ether, separated by thin-layer chromatography, and quantified by liquid scintillation counting. Blank values were determined in the absence of cell lysate under identical conditions and subtracted.

##### Statistical Analysis

Data are presented as mean values ± S.E. of *n* experiments. Individual concentration-response curves were fitted to a Hill-type model giving estimates of GTN potency (EC_50_) and efficacy (*E*_max_). Analysis of variance with post hoc Bonferroni-Dunn test was used for comparison between groups by using StatView® (Version 5.0). Significance was assumed at *p* < 0.05.

## Author Contributions

M. O., E. E., and M. W. W. conducted experiments and analyzed the data. M. R. and D. K. contributed new reagents/analytical tools. R. M., W. F. G., J. T. F., A. S., and B. M. conceived and designed the study. M. O. and B. M. wrote the manuscript.

## Supplementary Material

Supplemental Data

## Footnotes

2 The abbreviations used are:

GTN: glycerol trinitrate (nitroglycerin)
GDN: glycerol dinitrate
AdV: adenovirus
ALDH2: aldehyde dehydrogenase-2
C-geNOp: cyan fluorescent genetically encoded NO probe
DEA/NO: 2,2-diethyl-1-nitroso-oxyhydrazine
DTPA: diethylene triamine pentaacetic acid
NOC-7: 3-(2-hydroxy-1-methyl-2-nitrosohydrazino)-*N*-methyl-1-propanamine
sGC: soluble guanylate cyclase
SOD: superoxide dismutase
VSMC: vascular smooth muscle cell(s)
MOI: multiplicity of infection.
