# Supplementary material for: Formation of Nitric Oxide by Aldehyde Dehydrogenase-2 Is Necessary and Sufficient for Vascular Bioactivation of Nitroglycerin
Source: J Biol Chem. 2016 Sep 27;291(46):24076–84. doi: 10.1074/jbc.M116.752071 (PMC5104933; doi:10.1074/jbc.M116.752071)
Supplement: Supplemental Data [file supp_291_46_24076__index.html]

Formation of Nitric Oxide by Aldehyde Dehydrogenase-2 Is Necessary and Sufficient for Vascular Bioactivation of Nitroglycerin — NO Formation Is Essential for GTN Bioactivity — Supplemental Data 

# Formation of Nitric Oxide by Aldehyde Dehydrogenase-2 Is Necessary and Sufficient for Vascular Bioactivation of Nitroglycerin

## Supplemental Data

- supplemental figures (.pdf, 3.3 MB) - additional data obtained with the NO sensor
