# Supplementary material for: Formation of Nitric Oxide by Aldehyde Dehydrogenase-2 Is Necessary and Sufficient for Vascular Bioactivation of Nitroglycerin
Source: J Biol Chem. 2016 Sep 27;291(46):24076–84. doi: 10.1074/jbc.M116.752071 (PMC5104933; doi:10.1074/jbc.M116.752071)
Supplement: Supplemental Data [file 10.1074_M116.752071_jbc.M116.752071-1.pdf]

SUPPLEMENTAL DATA

Formation of nitric oxide by aldehyde dehydrogenase-2 is necessary and sufficient for vascular bioactivation of nitroglycerin

Marissa Opelt[1], Emrah Eroglu[2], Markus Waldeck-Weiermair[2], Michael Russwurm[3], Doris Koesling[3], Roland Malli[2], Wolfgang F. Graier[2], John T. Fassett[1], Astrid Schrammel[1], and Bernd Mayer[1]

[1]Institute of Pharmaceutical Sciences, Department of Pharmacology and Toxicology, University of Graz, Austria; [2]Institute of Molecular Biology and Biochemistry, Center of Molecular Medicine, Medical University of Graz, Austria; [3]Department of Pharmacology and Toxicology, Ruhr University Bochum, Germany

To whom correspondence should be addressed: Dr. Bernd Mayer, Department of Pharmacology and Toxicology, University of Graz, Humboldtstrasse 46/I, A-8010 Graz, Austria, Telephone: +43-316-380-5567, FAX: +43-316-380-9890, E-mail: mayer@uni-graz.at

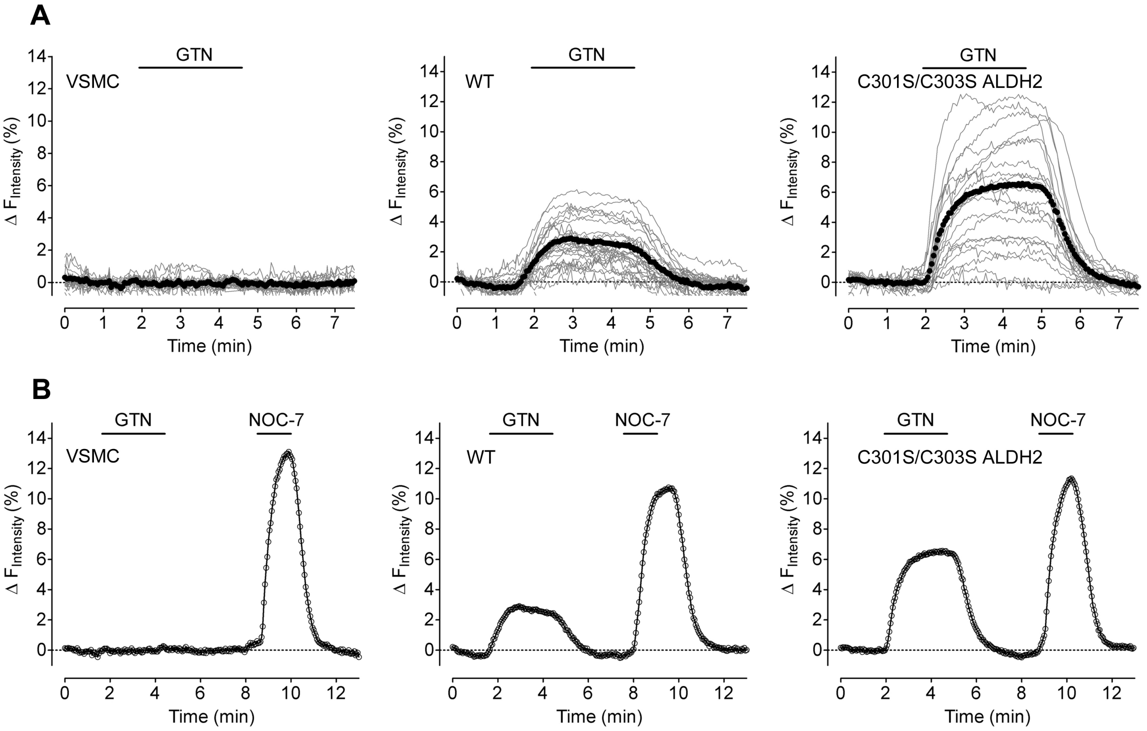

Supplementary Figure 1. Live-cell imaging of NO release induced by GTN or NOC-7 in vascular smooth muscle cells. A, Individual traces of NO release over time were measured in VSMC infected with C-geNOp alone (left panel) or in combination with either WT (middle panel) or C301S/C303S ALDH2 (right panel) in response to 1 µM GTN in the presence of 1 mM DTT by live-cell imaging as described in *Experimental Procedures.* Average curves with SEM are shown in Figure 2B (main text). **B,** Cellular NO release of VSMC expressing either C-geNOp alone (left panel) or in combination with either WT (middle panel) or C301S/C303S ALDH2 (right panel) in response to 1 µM GTN or 10 µM NOC-7 in the presence of 1 mM DTT (n=27 for VSMC; n=26 for VSMC+WT; n=20 for VSMC+ C301S/C303S ALDH2). Data are expressed as inverted curves (1-F/F0 in %) of the number of experiments indicated in the panel description above.

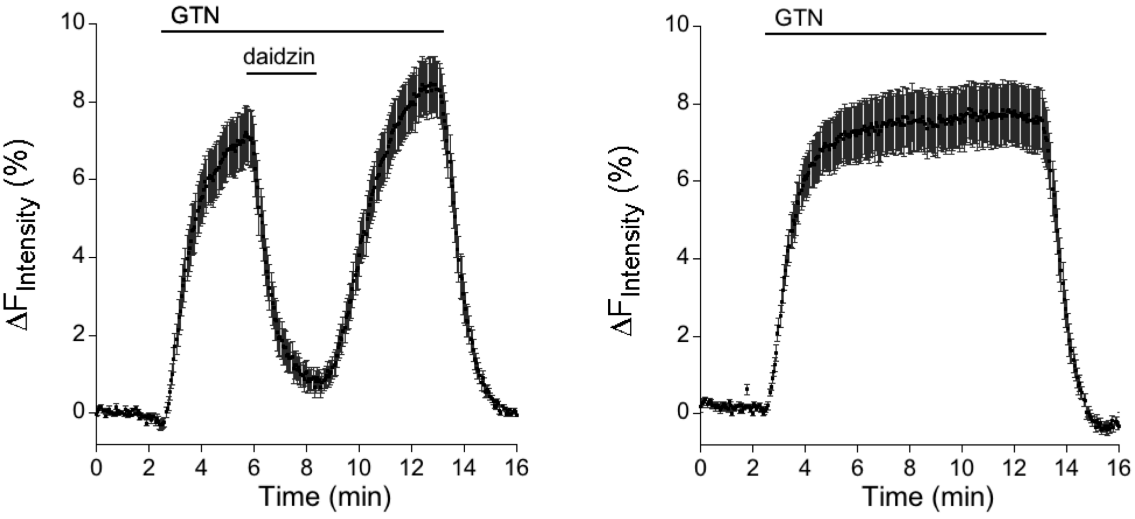

Supplementary Figure 2. Effect of daidzin on GTN-derived NO formation in vascular smooth muscle cells expressing C301S/C303S ALDH2. Average curves showing NO release over time in response to 1 µM GTN in VSMC expressing C301S/C303S ALDH2 under control conditions (right panel; n=16) or upon application and subsequent washout of 0.2 mM daidzin (left panel; n=19). Data are expressed as inverted curves (1-F/F0 in %).

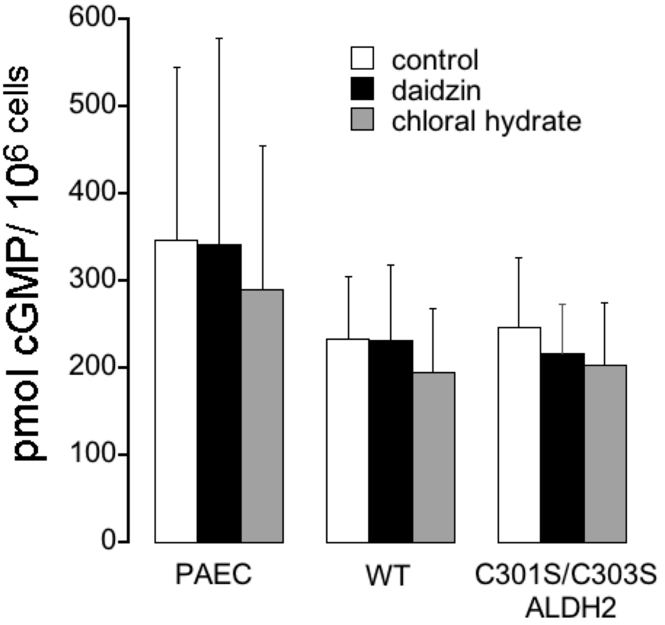

Supplementary Figure 3. GTN-induced cGMP accumulation in cultured porcine aortic endothelial cells. Non-infected and infected (WT or C301S/C303S ALDH2) porcine aortic endothelial cells were incubated with 1 μM DEA/NO in the absence and presence of 0.4 mM daidzin and 5 mM chloral hydrate and cGMP formation was determined as described in *Experimental Procedures.* Data represent mean values ± SEM of three to five independent experiments.
